# Mediterranean Species of the Spittlebug Genus *Philaenus*: Modes of Chromosome Evolution

**DOI:** 10.1673/031.012.5401

**Published:** 2012-04-17

**Authors:** Anna Maryańska-Nadachowska, Valentina G. Kuznetsova, Dorota Lachowska, Sakis Drosopoulos

**Affiliations:** ^1^ Institute of Systematics and Evolution of Animals, Polish Academy of Sciences, Poland; ^2^ Zoological Institute, Russian Academy of Sciences, Russia; ^3^ Department of Entomology, Institute of Zoology, Jagiellonian University, Poland; ^4^ Agricultural University, Athens, Greece

**Keywords:** cytogenetics, karyotype evolution, sex chromosome system

## Abstract

The evolution of karyotypes and sex determination system of *Philaenus* Stål (Auchenorrhyncha: Aphrophoridae) species is studied here in detail. The most plausible scenario of chromosomal rearrangements accompanying phylogenetic differentiation in *Philaenus* is advanced. It is postulated that the ancestral karyotype of *Philaenus* was 2n = 24 + X0. Karyotype changes occurred several times independently in the genus. The karyotype of 2n = 22 + X0 (*P. spumarius* and *P. tesselatus*) originated from 2n = 24 + X0 by fusion between two autosomal pairs. The neo—XY system (*P. arslani, P. loukasi, P. signatus, P. maghresignus,* and *P. tarifa*) also originated from the 24 + X0 karyotype by means of independent fusions between autosomes and the original X chromosome. The neo—X_1_X_2_Y system (*P. italosignus*) evolved from the 2n = 22 + neo—XY karyotype by an additional fusion between the Y chromosome and one more autosomal pair. The neo—X_n_Y system of *P. italosignus* is the first reported case of an evolutionarily fixed multiple sex chromosome system in Auchenorrhyncha.

## Introduction

The spittlebug genus *Philaenus* Stål (Auchenorrhyncha: Aphrophoridae) has long attracted the particular interest of biologists due to its high color polymorphism. The nature and origin of this polymorphism and its possible contribution to the evolution of reproductive isolation and sympatric speciation have been extensively documented for *P. spumarius.* This species is widely distributed, covering most of the Palaearctic region and extending into the Nearctic, as well as most other temperate regions of the earth and many oceanic islands (Halkka and Halkka 1990; Stewart and Lees 1996; Drosopoulos 2003; Drosopoulos et al. 2010). *Philaenus spumarius* is a highly polyphagous insect, and has become a pest of fodder plants and strawberries in areas where it is not a native species (Halkka et al. 1967; [Bibr bibr58]). Due to outstanding polymorphism in adult dorsal color/pattern, more than 50 synonyms have been given to *P. spumarius* (Nast 1972). Until the late 1980s, only three species were recognized in the genus *Philaenus:* the Holarctic *P. spumarius,* the Mediterranean species *P. signatus* (which inhabits the Balkans and Middle East), and *P. tesselatus* (southern Iberia and Maghreb). *Philaenus tesselatus* was often treated as a subspecies (Wagner 1959) or a synonym of *P. spumarius* (Nast 1972). However, more recent studies suggested that it is a valid species distinct from *P. spumarius* (Drosopoulos and Quartau 2002). Since the 1990s, as a result of purposeful morphological studies on *Philaenus* in the Mediterranean region, five further species have been described: *P. loukasi* (southern Balkans), *P. arslani* (Middle East), *P. maghresignus* (Maghreb and southern Spain), *P. italosignus* (southern Italy and Sicily), and *P. tarifa* (southern Iberia).

The Mediterranean species of *Philaenus* were shown to be sympatric with *P. spumarius,* while partly allopatric with each other. At present, eight species are recognized in the genus *Philaenus.* The current taxonomy of this genus accepts a division of these species into two groups based on morphological similarities in the male anal tube: the *“spumarius”* group (*P. spumarius, P. tesselatus, P. loukasi,* and *P. arslani*), and the *“signatus”* group (*P. signatus, P. italosignus, P. maghresignus,* and *P. tarifa*) (Drosopoulos and Remane 2000). According to larval food plant preferences, the genus is subclassified into the three groups developing: (1) on the lily, *Asphodelus aestivus* (= *A. microcarpus*) (*P. signatus, P. italosignus, P. maghresignus,* and *P. tarifa*), (2) on xerophilic plants (*P. loukasi* and *P. arslani*), and (3) on various dicotyledonous and monocotyledonous plants (*P. spumarius* and *P. tesselatus*) (Drosopoulos 2003). The results of a recent phylogenetic study of *Philaenus* using nucleotide sequences from two mitochondrial (COI and CytB) genes and one nuclear (ITS2) region is in general agreement both with morphological and food plant preference classifications, with the exception of *P. maghresignus,* placed as a sister taxon to all remaining *Philaenus* species (Maryańska-Nadachowska et al. 2010).

Over 90% of speciation events are suggested to be accompanied with chromosomal rearrangements (White 1978). Auchenorrhyncha possess holokinetic chromosomes; that is, their chromosomes do not have a primary constriction (the centromere) (Halkka 1959). Because of the absence of the centromere as a morphological marker, and also because of the paucity of convenient differential techniques, the interchromosomal (particularly intrachromosomal) rearrangements cannot be detected in holokinetic chromosomes. These chromosomes have thus no distinctive features for individual identification in a karyotype, besides size differences, if present. In holokinetic chromosomes, a kinetochor plate (to which the spindle microtubules attach) covers all or the majority of the chromosome surface (Wolf 1996). Theoretically, the large kinetochor plate facilitates karyotype evolution by means of fusion and fission of holokinetic chromosomes, since on the one hand there is no risk of the formation of dicentric chromosomes and, on the other hand, even relatively small chromosome fragments can have a part of the kinetochore plate and thus be attached to the spindle. For the reasons mentioned above, these rearrangements are conventionally accepted as the most common mechanisms of chromosome evolution in holokinetic groups. However, contrary to what may be expected, Auchenorrhyncha seem to be characterized by stable or only slightly variable karyotypes at the levels of genera, tribes, and families (Halkka 1959; Kirillova 1986, 1988; Emeljanov and Kirillova 1989, 1991) suggesting that chromosomal fusions/fissions have not played a key role in karyotype evolution and speciation within this group. For example, almost all species of the tribes Issini (Issidae) and Almanini (Dictyopharidae) were found to have 2n = 26 + XX/X0 (Maryańska-Nadachowska et al. 2006; Kuznetsova et al. 2010) and 2n = 24 + neo— XY (Kuznetsova et al. 1986, 2009a), respectively.

Theoretically, in holokinetic chromosomes, rearrangements can be detected if advanced techniques of molecular cytogenetics are used to establish chromosomal markers. However, the most informative techniques, such as immunofluorescence, chromosome painting, genomic in situ hybridization (GISH), and FISH mapping of genes, which are currently used in some economically important holokinetic organisms (Mandrioli et al. 2003; Mandrioli and Borsatti 2007; Marec et al. 2010), are not yet developed or available for Auchenorrhyncha (Kuznetsova et al. 2010). Over the past several decades, a number of studies have used conventional banding techniques (C-, AgNOR-, DAPI/CMA_3_banding) for the study of auchenorrhynchan karyotypes (Noda and Tatewaki 1990; Perepelov et al. 2002; Kuznetsova et al. 2003, 2009a, 2009b, 2010; Maryańska-Nadachowska et al. 2006); however, these studies developed only a few chromosomal markers. Additionally, their limited taxonomic representation failed to provide comprehensive insight into the comparative cytogenetics of the group (Kuznetsova et al. 2010).

The family Aphrophoridae, to which the genus *Philaenus* belongs, is a group with fairly diversified karyotypes. In 29 studied species assigned to nine genera within Aphrophoridae, the number of autosomes ranges from 11 to 30 including all possible values of the diploid number (Kirillova 1986; Kuznetsova et al. 2003; Maryańska-Nadachowska et al. 2008, 2010). Almost without exception, the evidence today concerns just the number of chromosomes and the type of sex determination, the data being too few in number in each genus studied for any conclusions to be reached. In the comparatively better—studied genus *Aphrophora,* all species (probably with the only exception of *A. quadrinotata*) have 28 autosomes in the diploid complement. In contrast, the genus *Philaenus* demonstrates a wide variety of different karyotypes that have been briefly described by Maryańska-Nadachowska et al. (2010). At the present time, *P. spumarius* and *P. arslani* are the only two aphrophorid species comprehensively studied using chromosome banding techniques (Kuznetsova et al. 2003; Maryańska-Nadachowska et al. 2008).

The aims of this study were to apply these techniques to the six other *Philaenus* species and used cytogenetic markers for understanding the taxonomy and intrageneric relationships of the *Philaenus* and elucidating the modes of chromosomal rearrangements in the evolution of the genus.

## Materials and Methods

Table 1 lists species, dates and collection localities, host plants, and number of specimens studied. Insects were fixed in Carnoy, a mixture of 96% alcohol and glacial acetic acid (3:1), and stored in fixative at 4 ^°^C until slides were made. Air—dried chromosome preparations were made by squashing testicular follicles in 45% acetic acid and freezing in dry ice. The coverslips were then taken off with a razor blade. The techniques of chromosome staining followed Kuznetsova et al. (2003) and Maryańska-Nadachowska et al. (2008): conventional Feulgen-Giemsa staining for the visualisation of standard karyotypes, silver—staining for the visualization of the nucleolus organizing regions (NORs), and C—banding for the detection of the constitutive heterochromatic regions (C—heterochromatin). In order to reveal the molecular composition of C—bands, some slides were stained with base specific fluorochromes CMA_3_ and DAPI. For methodological details see Kuznetsova et al. (2003). Analysis of slides was performed using a Nikon Eclipse E400 light microscope (www.nikon.com) at 1000× magnification. Photomicrographs were taken using a Nikon DS-U1 camera. All voucher specimens are preserved in the Institute of Systematics and Evolution of Animals, Polish Academy of Sciences in Kraków, Poland.

**Table 1.**
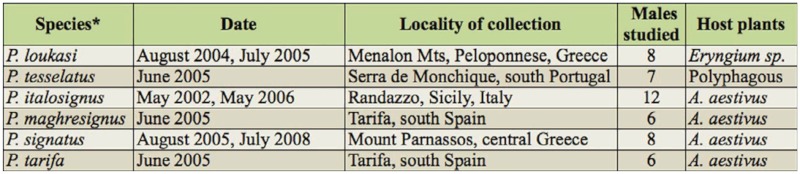
Species, collection localities, number of specimens, and host plants of six *Philaenus* species studied.

## Results

### The “*spumarius*” group

Published data: *P. spumarius*: 2n = 23 (22 + X0) (Kuznetsova et al. 2003); *P. arslani*: 2n = 20 (18 + neo—XY) (MaryańskaNadachowska et al. 2008).

### *Philaenus tesselatus*: 2n = 23 (22 + X0)

**Figure 1a–g.**
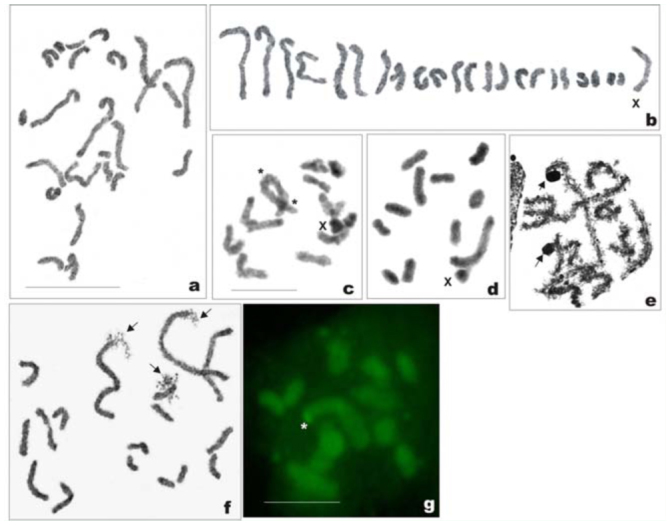
Mitotic and meiotic chromosomes of *Philaenus tesselatus.* (a) mitotic prometaphase; (b) karyogram of mitotic prometaphase; (c) C—banded diakinesis (asterisks indicate two chiasmata in the largest bivalent); (d) C—banded metaphase l; (e) Ag—stained diplotene (arrows indicate NORs); (f) incomplete mitotic prometaphase with NORs—bearing chromosomes (arrows); (g) CMA_3_treated metaphase 1 with one positive signal on the largest bivalent (asterisk). Bar = 10 µm. Scale bar on (a) refers to (a), (b), and (f); scale bar on (c) refers to (c), (d), and (e). High quality figures are available online.

The male mitotic complement was composed of 23 chromosomes which gradually decrease in size, with the X chromosome close in size to one of the longer pairs of autosomes (probably AA_3_) (Figures 1a, 1b). All of the chromosomes had a well—defined holokinetic structure without visible constrictions (the centromeres). In meiosis, 11 bivalents, one clearly larger than the others, and a univalent X chromosome were observed (Figures 1c, 1d). Noteworthy was the high level of condensation of the X in meiosis. The bivalents displayed one or occasionally two (in larger bivalents; Figure 1c) terminal/subterminal chiasmata. Apparently after C—banding, the karyotype was characterized by a small amount of constitutive heterochromatin (C— heterochromatin) (Figures 1c, 1d). At diakinesis and metaphase I (MI), small subtelomeric C—bands were visible on larger bivalents and on the X (Figures 1c, 1d). In the silver—stained mitotic (Figure 1e) and meiotic (Figure 1f) nuclei, masses of argyrophylic material (indicative of NORs) were more often revealed on the largest (AA_1_) and on one of the middle—sized autosomal pairs. The CMA_3_ treatment revealed GC—rich regions (probably corresponding to NORs) only on AA1 (Figure 1g).

### *Philaenus loukasi*: 2n = 20 (18 + neo—XY)

**Figure 2a–f.**
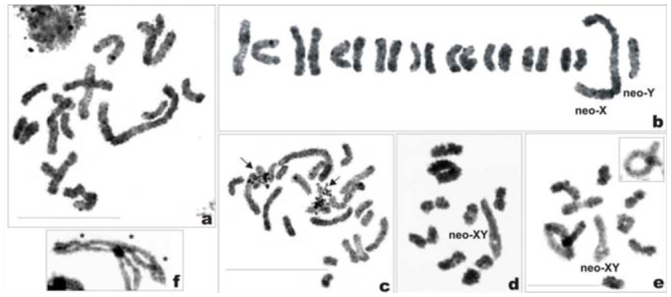
Mitotic and meiotic chromosomes of *Philaenus loukasi.* (a) mitotic metaphase; (b) karyogram of mitotic metaphase; (c) Ag—stained mitotic metaphase (arrows indicate two clusters of argyrophilic material); (d) diakinesis; (e) C—banded diakinesis, in the larger autosomal bivalent two terminal chiasmata are visible; in small frame the largest autosomal bivalent with terminal and interstitial chiasmata from another plate is added; (f) diplotene, bivalent with three chiasmata (asterisks). Bar = 10 µm. Scale bar on (a) refers to (a) and (b); scale bar on (e) refers to (d), (e), and (f). High quality figures are available online.

The male mitotic complement was composed of 20 chromosomes, including: 18 autosomes, which more or less gradually decrease in size; the Y chromosome, close in size to the medium—sized autosomal pairs; and the X chromosome, which was markedly longer than the AA_1_; the X was approximately three times longer than the Y (Figures 2a, 2b). This suggests a neo—XY sex determination. Silver impregnation showed that NOR sites were located on autosomes; however, we were unable to detect the number of NOR—bearing autosomes (one or two pairs) at the mitotic prometaphase (Figure 2c). At diakinesis, 10 bivalents were observed (Figures 2d, 2e). All the bivalents, including the heteromorphic XY bivalent, displayed one terminal chiasma each, except for AA^1^, which sometimes showed two or even three chiasmata in different locations (Figures 2d–2f). The marked heteromorphism of the XY pair and the presence of chiasma were indicative of the neo—XY type. C— banding induced prominent C—positive bands, both terminal and interstitial, in almost every bivalent (Figure 2e).

### The *“signatus”* group

Published data: absent.

### *Philaenus signatus:* 2n = 24 (22 + neo—XY)

**Figure 3a–h.**
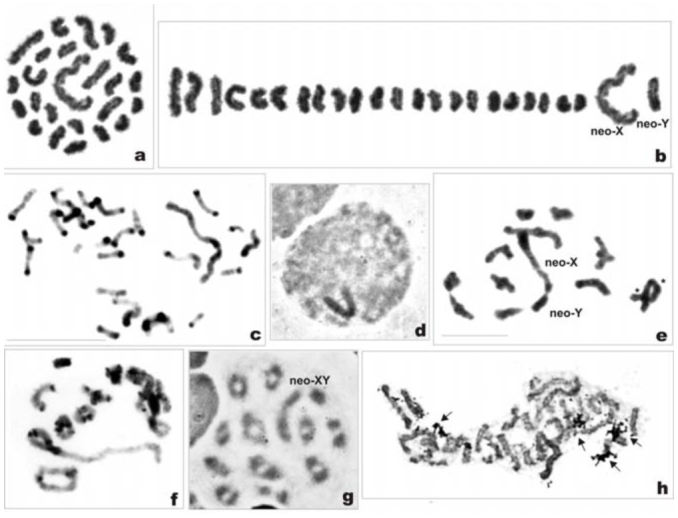
Mitotic and meiotic chromosomes of *Philaenus* *signatus.* (a) mitotic metaphase; (b) karyogram of mitotic metaphase; (c) C—banded mitotic prometaphase; (d) interphase, note the neo—X chromosome as a long heteropycnotic body; (e) diakinesis, bivalents are connected by terminal or interstitial chiasmata, asterik indicate two chiasmata in the largest bivalent; (f) C—banded diakinesis; (g) metaphase I; (h) silver—stained mitotic prometaphase (arrows indicate four clusters of argyrophilic material connected to chromosomes other than sex chromosomes). Bar = 10 µm. Scale bar on (c) refers to (a), (b), and (c); scale bar on (e) refers to (d–h). High quality figures are available online.

In spermatogonial metaphases, 24 chromosomes were observed, among which were the very long X and five medium—sized ones (pairs AA_1_ and AA_2_, and the Y). All remaining chromosomes (pairs AA_3_-AA_11_) gradually decreased in size (Figures 3a–b). The sex determination system is suggested to be of a neo—XY type. The neo—X was much longer than AA_1_ and three times longer than the neo—Y. The neo—X chromosome was visible in the interphase nuclei as a long heteropycnotic body (Figure 3d). At diakinesis, there were 11 autosomal bivalents with 1–2 chiasmata each, and an XY sex bivalent, which was very large and highly heteromorphic. The neo—X (the original X + fused autosome) and neo—Y (the other homologue of the fused autosome) chromosomes were connected by a terminal chiasma. A part of the neo—X was heteropycnotic at this stage, and the chiasma in the sex bivalent was always formed at a point opposite to this part (Figures 3e–g). As revealed by C—banding, the species displayed a fairly large amount of heterochromatin, located as prominent bands on one telomere (but more often on both telomeres) of every chromosome (Figures 3c, 3f). Silver impregnation disclosed four argyrophilic areas in mitotic prometaphase nuclei (Figure 3h). Argyrophilic material was connected to chromosomes other than the neo—X (this latter is easily identified at this stage), suggesting the presence of two pairs of NOR—bearing autosomes, though these failed to be identified.

### *Philaenus tarifa*: 2n = 24 (22 + neo—XY)

**Figure 4a–e.**
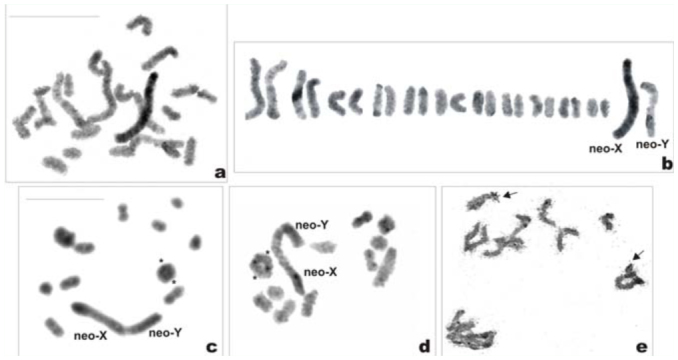
Mitotic and meiotic chromosomes of *Philaenus tarifa.* (a) mitotic metaphase; (b) karyogram of mitotic metaphase; (c) diakinesis, bivalents with one or two chiasmata, asterisks indicate two chiasmata in a large bivalent; (d) C—banded diakinesis; (e) Ag—stained diplotene, arrows indicate two bivalents bearing NORs. Bar = 10 µm. Scale bar on (a) refers to (a) and (b); scale bar on (c) refers to (c), (d), and (e). High quality figures are available online.

Male mitotic prometaphase showed 24 chromosomes including a very long X and five medium—sized chromosomes (pairs AA_1_ and AA_2_, and the Y). All remaining autosomes (pairs AA_3_-AA_11_) gradually decreased in size. The sex determination system was of the neo—XY type (as in *P. signatus).* However, the size difference between the neo—X and AA_1_, as well as between the neo—X and the neo—Y, was not as marked as that in *P. signatus.* The neo—Y represented about 70% of the neo—X chromosome length (Figures 4a, 4b). At diakinesis, there were 11 autosomal bivalents with 1–2 chiasmata each, and the neo—XY sex bivalent, which was very large and heteromorphic. Neo—X and neo—Y chromosomes were connected by a terminal chiasma. A part of the neo—X (the original X) was heteropycnotic at this stage, and the chiasma in the sex bivalent was always formed at the point opposite to this part (Figure 4c, d). C—banding induced dot—like, faintly discernible bands on several bivalents, indicative of a small amount of constitutive heterochromatin in the complement (Figure 4d). Silver impregnation showed that two autosomal bivalents had NORs; however, these bivalents failed to be identified (Figure 4e).

### *Philaenus maghresignus:* 2n = 24 (22 + neo— XY)

**Figure 5a–d.**
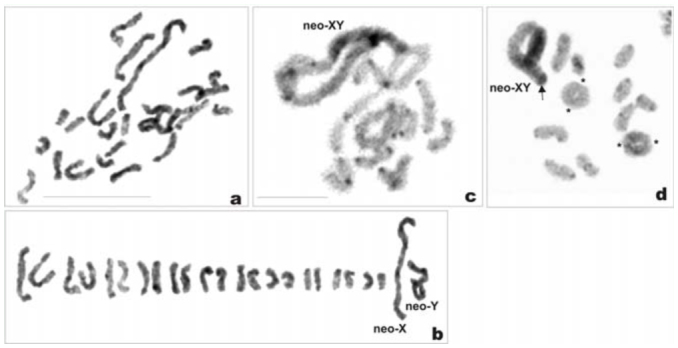
Mitotic and meiotic chromosomes of *Philaenus maghresignus.* (a) mitotic metaphase; (b) karyogram of mitotic metaphase; (c) C—banded diplotene; (d) C—banded diakinesis, one telomere of the neo—X is marked with a large block of heterochromatin (arrow), asterisks indicate two chiasmata in large autosomal bivalents. Bar = 10 µm. Scale bar on (a) refers to (a) and (b); scale bar on (c) refers to (c) and (d). High quality figures are available online.

Male mitotic prometaphase revealed 24 chromosomes including the very long X and five medium—sized chromosomes (pairs AA_1_ and AA_2_ and the Y). All remaining autosomes (pairs AA_3_-AA_11_) showed a size gradient from large to small (Figures 5a, 5b). The sex determination system is suggested to be of a neo—XY type. The neo—X was much longer than the AA_1_ and approximately two times longer than the neo—Y, the latter being the second largest chromosome of the set. In diplotene/diakinesis, 11 autosomal bivalents with one or two chiasmata and the very large and heteromorphic neo—XY bivalent were observed. Neo—X and neo—Y chromosomes were connected by a terminal chiasma (Figures 5c, 5d). A part of the neo—X (the original X) was heteropycnotic at this stage. Noteworthy was the chiasma, which was always formed in the sex bivalent at the point opposite to the heteropycnotic part of the neo— X. C—heterochromatin was visible as small but prominent terminal bands in the majority of bivalents (Figures 5c, 5d). In the heteropycnotic part of the X chromosome, one telomere was marked with a large C—band (Figure 5d).

### *Philaenus italosignus:* 2n = 23 (20 + neo— neo—X_1_X_2_Y)

**Figure 6a–1.**
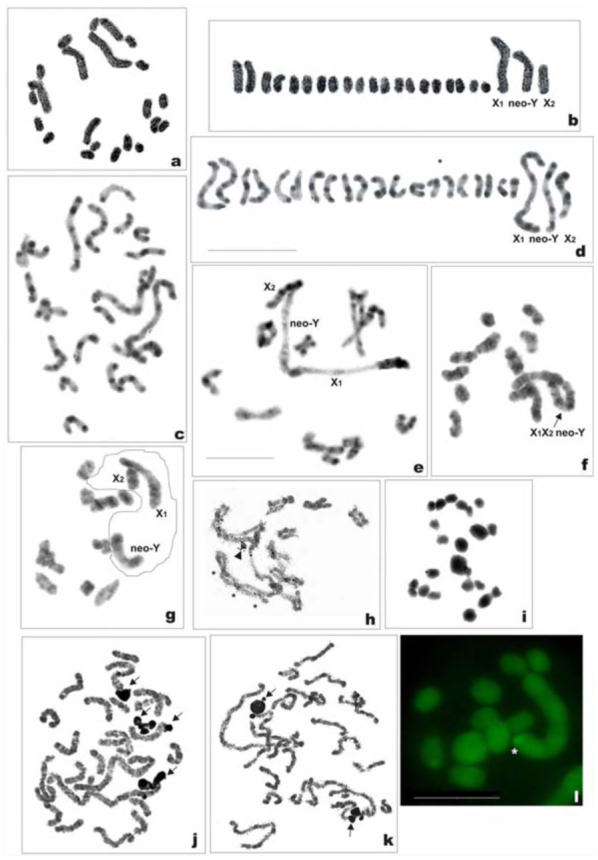
Mitotic and meiotic chromosomes of *Philaenus italosignus.* (a) mitotic metaphase; (b) karyogram of mitotic metaphase; (c) C—banded mitotic prometaphase; (d) C—banded karyogram of mitotic prometaphase; (e) and (f) C—banded diplotene/diakinesis, sex trivalent with chromosomes joint end— to—end in order: X_1_-Y-X_2_; (g) C—banded diakinesis, sex chromosomes appear as univalents (outlined); (h) Ag—stained diplotene, asterisk indicates three chiasmata in one bivalent; headarrow indicates NOR; (i) two daughter metaphases II with n = 10 + X_1_X_2_ and n = 10 + Y, respectively; (j) and (k) Ag—stained mitotic prometaphases with four and two NORs, respectively (arrows); (I) CMA_3_—treated metaphase I with one positive signal on the sex—chromosome trivalent (asterisk). Bar = 10 µm. Scale bar on (d) refers to (a–d); scale bar on (e) refers to (e–k). High quality figures are available online.

Spermatogonial metaphase showed 23 chromosomes including 20 autosomes and three sex chromosomes (Figures 6a–d). Based on meiotic stages, sex chromosomes were identified as X_1_, X_2_, and Y, and the sex determination system of this species is suggested to be of a neo—neo—XY type. The autosomes decreased in size from large to small. Sex chromosomes were different in size, with X_1_ and Y being the longest chromosomes of the set, and X_2_ was somewhat smaller than AA_1_. The X_1_ was about twice as long as X_2_, and the latter was about 1.5 times smaller than the Y. After standard staining, two pairs only (AA_1_ and AA_10_) could be easily distinguished among autosomes; the remaining autosomes were of similar size and could be arranged in pairs only arbitrarily (Figure 6b). This species displayed a great deal of C—heterochromatin (Figures 6c–g). Figures 6c and 6d (karyogram) show a C—banded early mitotic prometaphase, in which separate chromosomal pairs could be identified based on combined analysis of sizes and C—banding patterns. The pair AA_1_ and each sex chromosome had numerous prominent bands, both terminal and interstitial. Each member of AA_2_ showed terminal bands at ends, a subterminal band at one end, and a double band in the middle. The AA_3_ chromosomes displayed bands at ends, one terminal and the other subterminal. The remaining autosomes had three bands each, two terminal and one interstitial.

At diplotene/diakinesis, 10 autosomal bivalents and a trivalent of sex chromosomes were detected (Figures 6e–g). Bivalents generally had 1–2 chiasmata each; however, in larger bivalents three chiasmata sometimes formed (Figure 6h). In the sex trivalent, the X_1_, X_2_, and Y were joined end—to—end (probably by chiasmata) in the order: X_1_, Y, X_2_. In some diakinetic nuclei, sex chromosomes appeared as univalents (Figure 6g). As expected, two daughter metaphase II (MII) cells formed with n = 10 + X_1_X_2_ and 10 + Y, respectively (Figure 6i). Silver impregnation of mitotic nuclei revealed a variable number of chromosomes carrying argyrophilic material even in one male (Figures 6j, 6k). NOR—bearing chromosomes were unable to be identified at this stage. However, observation of silver—stained diplotene (Figure 6h) and MIs subjected to the GC—specific fluorochrome CMA_3_ treatment (Figure 6i) definitively showed the presence of an NOR on the sex trivalent.

## Discussion

### Characteristics of holokinetic chromosomes of *Philaenus*

The six Mediterranean species in this study, together with data concerning the worldwide *P. spumarius* and the Mediterranean *P. arslani* published earlier (Kuznetsova et al. 2003; Maryańska-Nadachowska et al. 2008), represent an exhaustive taxonomic sampling effort for *Philaenus.* In the genus *Philaenus,* four karyotype patterns have been described: 2n = 22 + X0 (*P. tesselatus* and *P spumarius*), 2n = 18 + neo—XY (*P. loukasi* and *P. arslani),* 2n = 22 + neo—XY (*P. signatus, P. maghresignus,* and *P. tarifa*), and 2n = 20 + X_1_X_2_Y (*P. italosignus*). Thus, the three values of autosome number (18, 20, 22) and the three types of sex determination (X0, neo—XY, and X_1_X_2_Y) appear characteristic of as few as eight *Philaenus* species. Such karyotypic diversity at the generic level is rare in Auchenorrhyncha (Kirillova 1986, 1988).

Conventional opinion holds that holokinetic chromosomes contain a small amount of constitutive heterochromatin, which is generally located on chromosome ends or in their vicinities (Blackman 1987). However, *Philaenus* species showed both terminal and interstitial C—bands on autosomes and sex chromosomes. The greatest amount of C— heterochromatin is found in *P. italosignus,* in which prominent C—bands are numerous and variably located along the complement, allowing the majority of homologous chromosomes to be identified. Thus, the present data agree with recent evidence from holokinetic animals (Kuznetsova et al. 1997, 2009b; Maryańska-Nadachowska 1999; Grozeva and Nokkala 2001; Golub et al. 2004; Angus et al. 2004; Pérez et al. 2005; Franco et al. 2006; Bressa et al. 2005, 2008) and plants (Collet and Westerman 1984; Sheikh and Kondo 1995; Vanzela and Guerra 2000; Guerra and Garcia 2004), suggesting that the amount and distribution of C— heterochromatin in holokinetic chromosomes are quite variable, as they are in monocentric chromosomes (Guerra et al. 2010; Kuznetsova et al. 2010).

The low number of chiasmata (estimated to be 1—2 from cytogenetic analyses) is a common pattern in the Auchenorrhyncha (Halkka 1964). It is suggested that this pattern represents one of the peculiar features of holokinetic bivalents, and as such is irrespective of the group as a whole (Nokkala et al. 2004). This assessment was founded on the detailed observations of the behavior of a three—chiasmatic bivalent during meiosis of *Baeopelma foersteri.* This bivalent was shown to be incapable of completing anaphase I because of its inability to resolve the chiasma located in its center. The authors attributed this to a specific condensation process inherent in holokinetic chromosomes. Inevitable elimination of cells with multiple chiasmata thus creates strong selection against the formation of more than two chiasmata in holokinetic bivalents. In our study, three and even four chiasmata were observed in larger bivalents of *P. loukasi* and *P. italosignus,* and previously in larger bivalents of *P. spumarius* (Kuznetsova et al. 2003) and *P. arslani* (Maryańska-Nadachowska et al. 2008), as well as in several other auchenorrhynchan species (Tian and Yuan 1997; Kuznetsova et al. 2009a, 2009b, 2010). Contrary to expectations, meiotic disturbances have never been observed in any of these cases, suggesting that the question of the number of chiasmata that can be successfully resolved in a holokinetic bivalent is still unresolved.

### Sex chromosome evolution in *Philaenus*

**Figure 7.**
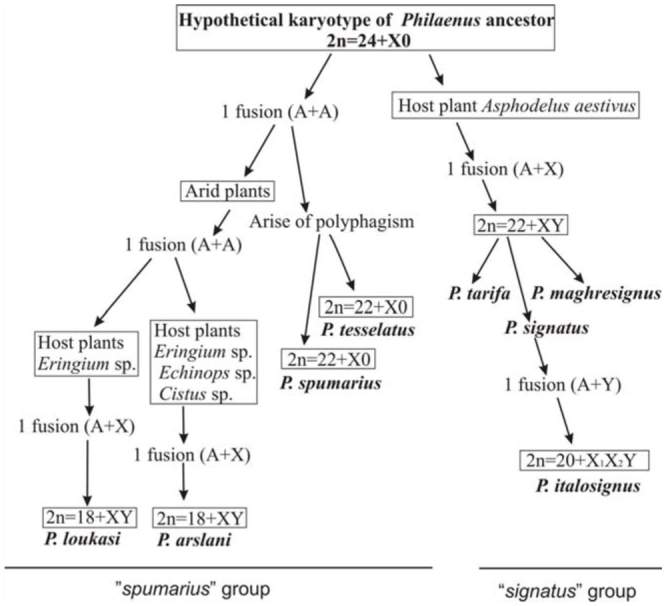
Presumable directions of chromosome rearrangements during karyotype evolution and changes of host plant preferences of *Philaenus* species. Karyotype alterations occurred several times independently in the genus. High quality figures are available online.

XX/X0 sex determination is of common occurrence in Auchenorrhyncha (Halkka 1959; Emeljanov and Kirillova 1990, 1992; Kuznetsova et al. 1998, 2009a, 2010; Kirillova 1986, 1988; Maryańska-Nadachowska et al. 2006), and almost certainly represents the ancestral type of sex determination in this group (Kuznetsova 1986) and in Hemiptera as a whole (Blackman 1995). It is very probable that ancestral karyotype of the genus *Philaenus* is 2n = 24 + X0 (Figure 7), or even 2n = 28 + X0 as in *Neophilaenus lineatus,* a representative of the most closely related genus (Halkka 1964; Kirillova 1986).

In the Auchenorrhyncha, only single species belonging to various genera are characterized by the neo—XY system. This type of sex determination usually arises from the X0 system as a result of fusion between the original X and an autosome, the homologue of the latter playing the role of the neo—Y and resulting in a lower number of autosomal pairs (Halkka 1959; Blackman 1995; Maryańska-Nadachowska et al. 2006; Kuznetsova et al. 2009a, 2010). The exceptions are species from the tribe Almanini (Dictyopharidae) belonging to 11 genera characterized by the neo—XY system (Kuznetsova 1986; Kuznetsova et al. 2009a).

Within the genus *Philaenus,* a neo—XY system is found in five species: *P. loukasi, P. arslani, P. signatus, P. maghresignus,* and *P. tarifa.* While these species share the same sex determination, they have a different number of autosomes: 18 in the first two (both from the *“spumarius”* group) and 22 in the three others (from the *“signatus”* group). However, the 2n = 20 + XY chromosomal set was not found in the genus. This can be attributed either to the extinction of species with this karyotype or, alternatively, to the existence of still unrecognized *Philaenus* species.

Clearly, the karyotype of 2n = 22 + neo—XY (inherent in *P. signatus, P. maghresignus,* and *P. tarifa*) could not have originated directly from 2n = 22 + X0. There are at least two possible explanations for the origin of this karyotype. It could gradually evolve through an autosomal fission resulting in 2n = 24 + X0 (with subsequent extinction of species with this karyotype) followed by an X—autosome fusion resulting in 2n = 22 + neo—XY. The other possibility is that the closest ancestor of the entire genus had already possessed 2n = 24 + X0, and this hypothesis appears more plausible (see Figure 7). *Philaenus italosignus* (2n = 20 + X_1_X_2_Y) demonstrates the next step of karyotype evolution within the *“signatus”* group. It is not difficult to explain the origin of the 2n = 20 + X_1_X_2_Y system of this species based on the way in which sex chromosomes associate at metaphase I of spermatogenesis (Figure 6e). In this case, the original neo—Y chromosome (in a complement with 2n = 22 + neo—XY) probably fused with the homologue of an autosomal pair resulting in the neo—neo— Y chromosome, the other homologue appeared as the X_2_ chromosome. The fused autosomal pair was most probably that one bearing an NOR, since the X_2_ in *P. italosignus* carries an NOR region. Interestingly, the neo— neo—Y (presumably including the homologue of the X_2_) did not carry an NOR in either silver impregnated or in CMA_3_ treated preparations. Within the Hemiptera, multiple sex chromosomes are very common in the Heteroptera (Ueshima 1979) and occasionally occur in Sternorrhyncha, namely in Aphidoidea (Hales 1989) and Coccoidea (Hughes-Schrader 1948). With a single exception recently described in the Heteroptera (Jacobs 2004), all multiple sex chromosome systems so far reported in these groups had arisen simply by fission of the original sex chromosomes into two or more pairs. Multiple X or Y chromosomes of this kind are characteristically smaller than the original ones, and there is no accompanying reduction in the number of autosomes (Blackman 1995). Until now only three hemipteran species, *Cacopsylla sorbi* and *C. mali* from Psylloidea (Sternorrhyncha) (Grozeva and Maryańska-Nadachowska 1995) and one *Austragalloides* sp. from the auchenorrhynchan family Cicadellidae (Whitten 1968), were reported to show multiple sex chromosome systems of the X_1_X_2_Y type originated by X—autosome fusions. However, in these species, the multiple systems occur in terms of sex chromosome polymorphism. Thus, the neo— X_n_Y system of *P. italosignus* is the first reported case of an autosomally—derived multiple sex chromosome system fixed at the species level within Auchenorrhyncha.

There is no doubt that neo—XY systems have evolved several times independently in the genus *Philaenus,* as evidenced by the available results. The neo—X and neo—Y chromosomes differ in size among species. The observed size differences confirmed that the neo—sex chromosomes appeared as a result of fusion between ancestral X and one of the autosomes. The X is the longest chromosome in each species. In *P. signatus* and *P. loukasi* (from the *“signatus”* and *“spumarius”* groups, respectively), the neo—X is approximately three times as long as the neo—Y, in *P. tarifa* and *P. maghresignus* (“*signatus”* group) it is only 1.5 times as long as neo—Y, and in *P. arslani (“spumarius”* group) neo—X and neo— Y chromosomes are approximately the same size. Thus, at least four independent translocation events have occurred in the evolution of the neo—XY in *Philaenus* species (Figure 7). These translocations clearly involved various autosomes of the ancestral chromosomal complement and resulted in the rise of the neo—X, which invariably exceeds the largest autosomes in size. In contrast, in the karyotype shared by *P. spumarius* and *P. tesselatus* (2n = 22 + X0), the X chromosome is noticeably smaller than the largest autosomes, supporting the occurrence of X— autosome fusions in the evolution of *Philaenus.* Moreover, based on the comparative size of neo—X and neo—Y chromosomes of the recent neo—XY species, we can preliminarily infer the particular fused autosomes in each translocation event. For example, in *P. arslani* (relatively small siz difference between sex chromosomes) fusion could have encompassed one of the larger (but not the largest, see below) autosomal pairs. In *P. tarifa* and *P. maghresignus* (larger difference in size between sex chromosomes) it was probably one of the middle—sized pairs, whereas in *P. signatus* and *P. loukasi* (largest size difference between sex chromosomes) the fusion involved one of the smaller pairs. In all cases (data are available only for *P. arslani, P. loukasi, P. tarifa,* and *P. signatus;* Maryańska-Nadachowska et al. 2008; this paper) the fused autosome was not the NOR—bearing one. This inference is based on the observation that NORs reside on autosomes in both X0 and neo—XY species. In two X0 species (*P. spumarius* and *P. tesselatus*) these autosomes are the largest and one of the medium—sized (probably 6^th^) pairs (Kuznetsova et al. 2003; this paper). In all of the neo—XY species, the sex determination system seems to be of quite recent origin, since the derived neo—Y chromosome is still homologous with the autosomal part of the neo—X, as evidenced by their chiasmatic connections in meiotic prophases.

## Conclusions

Numerous studies, each with a radically different approach, have been performed on the genus *Philaenus* to date. They have discussed a wide range of aspects such as morphology, distribution, host plant associations (Drosopoulos and Remane 2000; Drosopoulos 2003), molecular variation (Maryańska-Nadachowska et al. 2010) and cytogenetic characters (Kuznetsova et al. 2003; Maryańska-Nadachowska et al. 2008; this paper). For the reconstruction of phylogenetic relationships within *Philaenus,* mitochondrial and nuclear genetic markers were used (Maryańska-Nadachowska et al. 2010). Based on the topologies of all obtained trees, the monophyly of the genus was well supported, being congruent with morphological, ecological, and chromosomal data. The results confirmed the existence of three lineages within the genus. The first lineage included *P. maghresignus;* the second *P. tarifa, P. italosignus,* and *P. signatus;* and the third *P. loukasi, P. arslani, P. spumarius,* and *P. tesselatus.* The last clade, in turn, appeared to be subdivided into two further groups, one with *P. loukasi* and *P. arslani* and the other with *P. spumarius* and *P. tesselatus.* Taken together, molecular analyses and evidence from host—plant relationships and distribution patterns suggested that the ancestral *Philaenus* species may have used *Asphodelus aestivus* (the Mediterranean lily) as a host—plant, and this initial association remains characteristic of *P. maghresignus, P. tarifa, P. italosignus,* and *P. signatus. Philaenus loukasi* and *P. arslani* inhabit high altitudes feeding mostly on xerophilic plants, and this host—plant association is treated as a synapomorphic trait for these species, whereas the adaptation to a wide range of host—plants is a synapomorphy of *P. tesselatus* and *P. spumarius.* Regarding *P. spumarius,* this has been the leading factor promoting its postglacial expansion into temperate regions of Eurasia and other regions of the Palaearctic (Maryańska-Nadachowska et al. 2010).

We use the presumptions mentioned above to formulate the hypothesis that the changes of host—plant associations of *Philaenus* species would have to be accompanied by karyotype rearrangements (Figure 7). The lily species *A. aestivus* was suggested to come from middle Asia (Ayyad and Hilmy 1974; Rhizopoulou et al. 1997), and the nearest ancestor of *Philaenus* could have followed this plant into the Mediterranean region. This ancestor was likely to have had a karyotype of 2n = 24 + X0. An ancestral X0 sex determination system would allow the evolution of all other *Philaenus* karyotypes. There is a variety of ways in which this ancestral karyotype could be transformed into other karyotypes. We suggest that X—autosome fusions (at least two, each involving different autosomal pairs) first resulted in the rise of two different karyotypes of 2n = 22 + neo—XY (that of *P. maghresignus* and *P. tarifa,* on the one hand, and that of *P. signatus,* on the other) followed then by one further fusion between neo—Y and one more autosomal pair resulting in the karyotype of 2n = 20 + X_1_X_2_Y (inherent in *P. italosignus*). The above rearrangements are suggested to have occurred on *A. aestivus.* The following rearrangements took place during the chromosome evolution of *P. loukasi* and *P. arslani:* a fusion between two autosomal pairs (producing 2n = 20 + X0 of an unknown species) followed by two independent fusions, one between the original X and a small autosomal pair (giving the karyotype of *P. loukasi)* and the other between the original X and a larger autosomal pair (giving the karyotype of *P. arslani).* Finally, *P. tesselatus* and *P. spumarius* possibly originated from the ancestor by means of fusions of two autosomal pairs. It thus seems likely that at least eight autosome and autosome—sex chromosome fusions have occurred during the evolution of *Philaenus.*
